# Mapping Vulnerability to Potential Crisis Events in Surabaya City: A GIS-Based Approach

**DOI:** 10.12688/f1000research.145182.2

**Published:** 2024-07-05

**Authors:** Ali E. M. Jarghon, Nyoman Anita Damayanti, Inge Dhamanti, Hari Basuki Notobroto, Atik Choirul Hidajah, Anas M. M. Awad

**Affiliations:** 1 Faculty of public Health, Airlangga University, Surabaya, East Java, 60114, Indonesia; 2 Faculty of public Health, Airlangga University, Surabaya, East Java, 60114, Indonesia; 3 Faculty of public Health, Airlangga University, Surabaya, East Java, 60114, Indonesia; 4 Faculty of public Health, Airlangga University, Surabaya, East Java, 60114, Indonesia; 5 Faculty of public Health, Airlangga University, Surabaya, East Java, 60114, Indonesia; 6 faculty of Geodesy and Geomatics Engineering, Institut Teknologi Bandung, Bandung, West Java, 40132, Indonesia

**Keywords:** Vulnerability mapping, Disaster management, AHP, Public health preparedness, GIS-based analysis, GIS

## Abstract

**Background:**

This study aims to develop a vulnerability map for Surabaya using GIS-based Multi-Criteria Decision Analysis (MCDA) to assess the city’s vulnerability to COVID-19.

**Methods:**

Six key factors influencing vulnerability were identified and their relative importance determined through the Analytic Hierarchy Process (AHP) pairwise comparison matrix. GIS was utilized to classify Surabaya’s vulnerability into five levels: very low, low, medium, high, and very high.

**Results:**

The resulting vulnerability map provides essential insights for decision-makers, healthcare professionals, and disaster management teams. It enables strategic resource allocation, targeted interventions, and formulation of comprehensive response strategies tailored to specific needs of vulnerable districts.

**Conclusions:**

Through these measures, Surabaya can enhance its resilience and preparedness, ensuring the well-being of its residents in the face of potential emergency outbreaks.

## Introduction

In early 2020, countries worldwide faced susceptibility to the SARS-CoV-2 and COVID-19 coronaviruses. ^1^ The COVID-19 virus spread uncontrollably across the globe, evolving into a pandemic with severe health implications. ^2^ The COVID-19 outbreak had far-reaching effects on various aspects of daily life in numerous countries across the world. ^3^^,^^4^

The initial COVID-19 case in China, specifically in Wuhan, was identified. ^5^ Nonetheless, the virus propagated swiftly, and within a few months, confirmed cases had emerged in most countries worldwide. ^1^

Population may be at the greatest danger in the event of any crisis (such as the COVID-19 outbreak). ^6^^,^^7^ The city of Surabaya is the capital city of East Java Province, Indonesia, as well as the largest metropolitan city in the province. Surabaya is the second largest city in Indonesia after Jakarta. ^8^

The distribution of societal susceptibility to the impacts of a disaster is often spatial. ^9^^,^^10^ However, it’s essential to recognize that social vulnerability is a dynamic process significantly shaped by government initiatives and mitigation strategies. ^11^ Consequently, communities already facing vulnerability may experience an exacerbation of their situation due to an inadequate or delayed government response. ^11^

The term “vulnerability” describes a situation in which there is a potential for increased exposure to a community’s hazards. ^12^ Vulnerability mapping is a commonly utilized approach that suggests utilizing multiple determining factors to classify a particular community into various health vulnerability groups. ^13^ Population vulnerability significantly impacts crisis situations like the COVID-19 outbreak by influencing how communities are affected and how they can respond and recover. ^14^ Limited healthcare infrastructure, including access to medical facilities and resources, exacerbates these vulnerabilities. ^15^ Vulnerable populations are less able to prepare for, respond to, and recover from disasters. Understanding and addressing these vulnerabilities is crucial for effective crisis management and recovery strategies.

The concept of epidemic prediction mapping using multiple criteria analysis has been explored in several studies. ^16^ These studies often employ the multi-criteria decision analysis (MCDA) approach, considering numerous criteria in the vulnerability mapping of COVID-19. ^17^ Various factors, encompassing demographic (e.g., population), epidemiological (e.g., chronic diseases), and ecological/physical aspects (e.g., temperature), typically drive the mapping of COVID-19. ^10^^,^^18^^–^^20^ One of the most commonly employed MCDA strategies in these studies is the Analytic Hierarchy Process (AHP). ^21^ The AHP offers a systematic approach to assigning equitable weights to various influential criteria.

Recent research has employed Geographic Information Systems (GIS) and Multi-Criteria Decision Analysis (MCDA) to map and assess vulnerability to various crises, including pandemics. For instance, Shadeed and Alawna (2021) ^3^ utilized GIS-based vulnerability mapping in the West Bank, Palestine, to identify areas at higher risk of COVID-19 transmission. Similarly, Acharya and Porwal (2020) ^15^ developed a vulnerability index for managing and responding to COVID-19 in India, highlighting demographic, epidemiological, and ecological factors. These studies underscore the value of integrating spatial analysis with multi-criteria assessment to inform targeted public health interventions. This study addresses a GIS-based Multi-Criteria Decision Analysis (MCDA) framework to create a COVID-19 Vulnerability Index (CVI) specifically for Surabaya, Indonesia. This incorporates six critical factors: population size, population density, number of hospital beds, number of doctors, number of nurses, and availability of Intensive Care Units (ICUs) to produce a vulnerability map for Surabaya, enabling policymakers to identify high-risk areas and allocate resources more effectively.

A professional approach to mapping epidemic vulnerability and conducting risk assessment, such as for COVID-19 vulnerability, involves utilizing Geographic Information Systems (GIS) based Multi-Criteria Decision Analysis (MCDA). ^22^^–^^26^ In this study, the COVID-19 Vulnerability Index (CVI) was developed through the application of GIS-based Multi-Criteria Decision Analysis (MCDA). This index was then utilized to classify the governorates of Surabaya into different COVID-19 vulnerability categories.

This study aims to assess community vulnerability in emergency situations based on COVID-19 data. By establishing a COVID-19 vulnerability map for Surabaya city. Which is extremely valuable, Surabaya, a critical area due to its high population density and diverse socio-economic conditions, was chosen for this study to map vulnerabilities. These factors, along with its status as a major financial hub, necessitate understanding and mitigating potential crises. In addition, it helping decision-makers identify potential COVID-19 outbreaks and, in turn, implement appropriate mitigating strategies to protect public health, particularly in the governorates that are most at risk.

## Methods

### Study area

**Figure 1.**
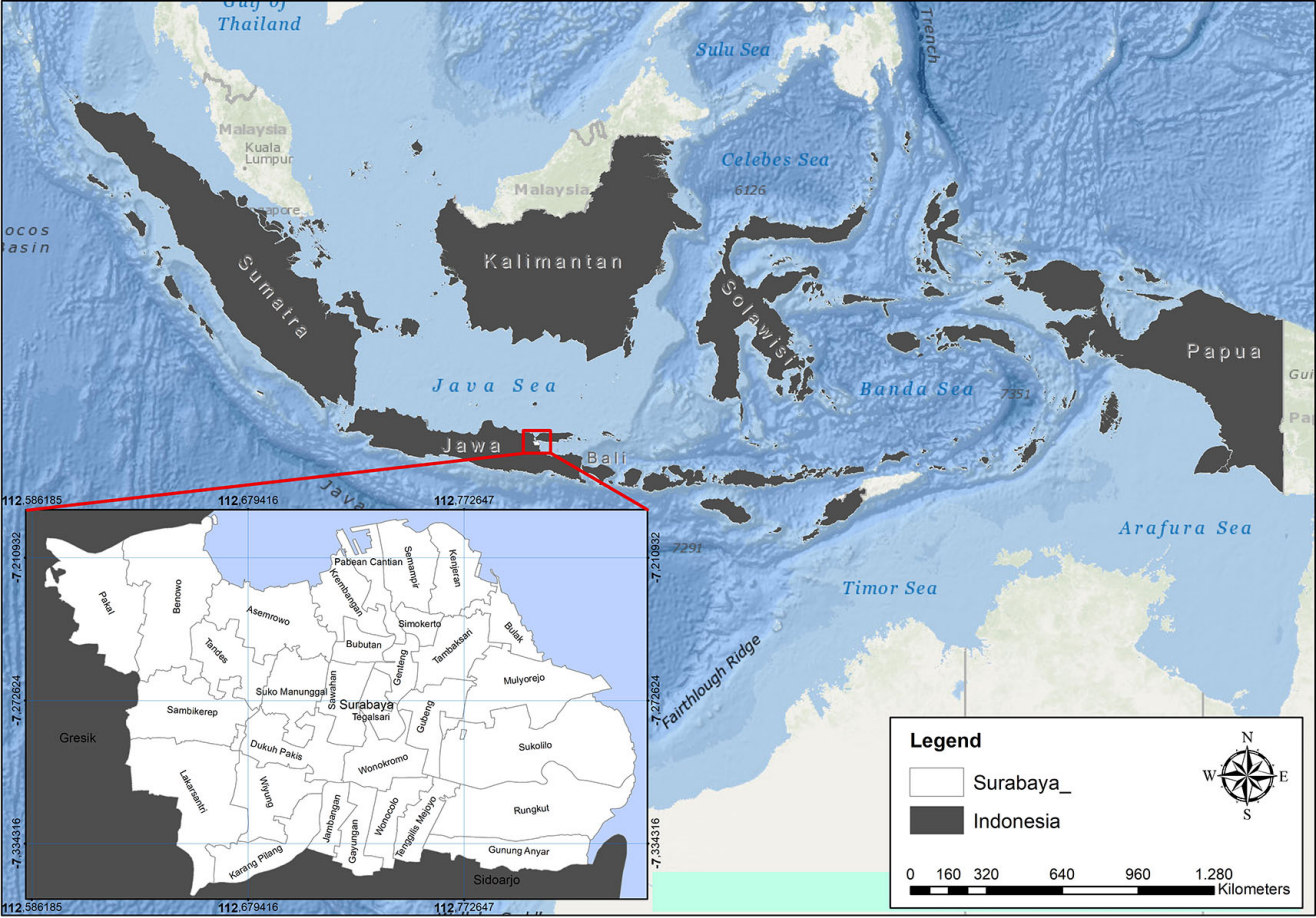
Location map of the City of Surabaya.

Surabaya is a city located in Indonesia. It is also the capital of the Jawa Timur province. The city is one of the most significant financial hubs in the country. As of the 2015 Census, the population of the city is 2.880.000. It is the second most populous city in Indonesia. The city proper contains a total surface area of 350.5 km ^2^ (135.3 sq mi). The metropolitan area however sprawls out to 5,925 km ^2^ (2,288 sq mi). The population density reaches upward of 9,900/km ^2^ (26,000/sq mi) in the city proper, and drops toward 2,200 per square kilometer (5,700 per square mile) as one moves toward the edge of the metropolitan area. The area of Surabaya City is divided into 5 regions (East, North, South, West, and Center) divided into 31 sub-districts and 163 villages see Figure 1 (Surabaya City Statistics Center, 2022).

### COVID-19 Vulnerability Index (CVI)

The COVID-19 vulnerability map in this study was constructed using the compiled CVI map. The design of the CVI map considered six crucial factors, as outlined in Table 1. These criteria were selected due to their capacity to increase COVID-19 vulnerability (P, PD, HB, D, N, and ICU) see Table 1.

**Table 1.** Criteria description.

	Criteria	Description	Source
1	Number of the population (P)	Total Percentage of Population	^8^
2	Population density (PD)	Population per km	^8^
3	Hospital beds (HB)	Number of hospital beds per district	^27^
4	No. Doctors (D)	Number of physicians per district	^27^
5	No. Nurses (N)	Number of nurses per district	^27^
6	No. ICU (ICU)	Number of ICU per district	^27^

### Data collection

The demographic data was obtained from the Surabaya City Statistics Center, while the healthcare infrastructure data was sourced from the Ministry of Health and the Regional Health Office.

**Figure 2.**
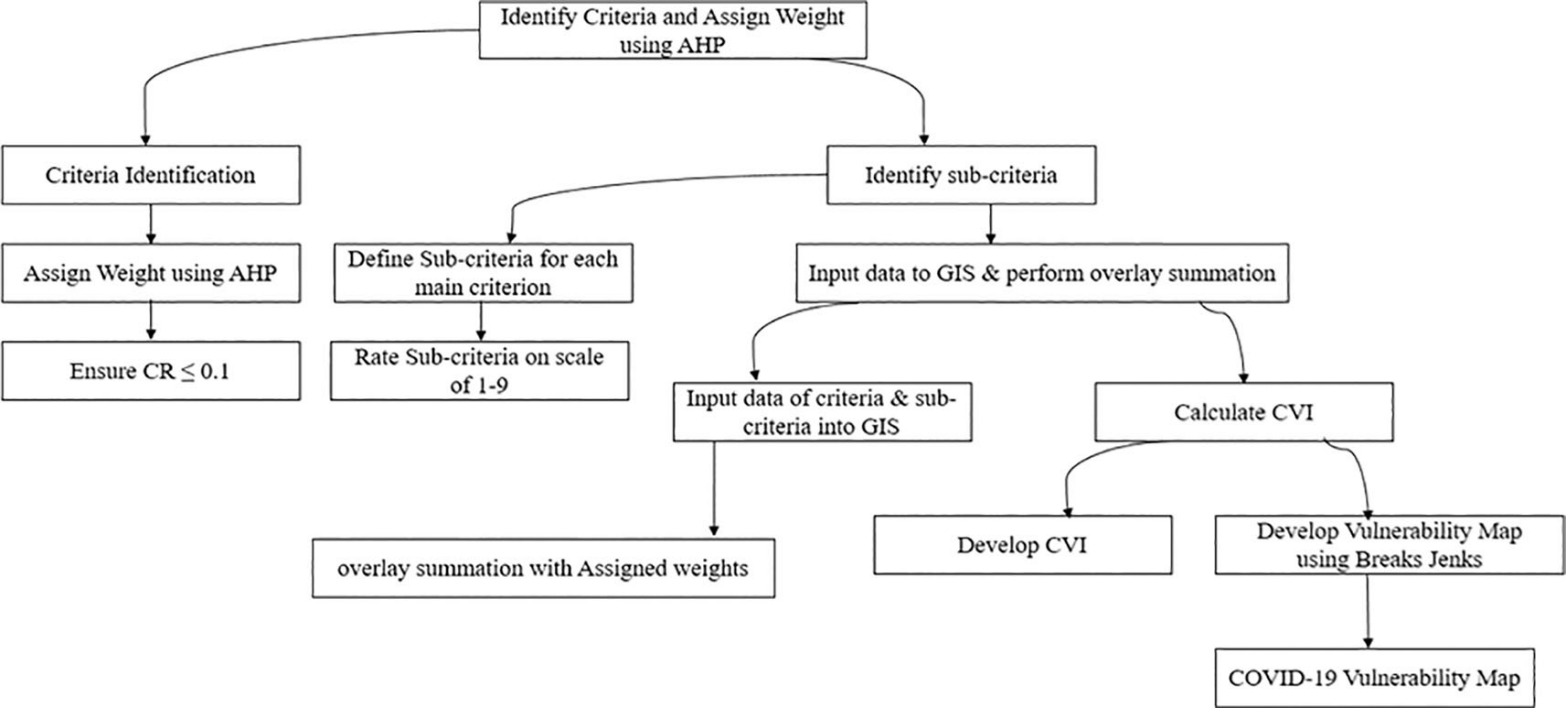
Methodological approach for developing of COVID-19 vulnerability map.

The overall methodological approach for developing the COVID-19 vulnerability map is detailed below. This includes the use of weighted overlay summation and the natural breaks (Jenks) method, as illustrated in Figure 2.

## Result

The AHP pairwise comparison matrix approach, presented in Table 2, was employed to allocate weights for the various CVI criteria. ^28^ Afterward, the consistency of these assigned weights was assessed by calculating a consistency ratio (CR) as follows. ^28^

$$\mathrm{CR}=\frac{\mathrm{CI}}{\mathrm{RI}}$$

$$\mathrm{CI}=\frac{\lambda -n}{n-1}$$

**Table 2.** CVI’s AHP pairwise comparison matrix.

Criteria	Population	Population density	Hospital beds	Doctor	Nurse	ICU	Weight
Population	1.0	0.3	2.0	3.0	3.0	4.0	0.21194
Population density	3.0	1.0	3.0	3.0	4.0	5.0	0.38447
Hospital beds	0.5	0.3	1.0	4.0	2.0	2.0	0.16136
Doctor	0.3	0.3	0.25	1.0	2.0	3.0	0.10548
Nurse	0.3	0.25	0.5	0.5	1.0	2.0	0.08771
ICU	0.25	0.2	0.5	0.3	0.5	1.0	0.05349

Abbreviation: ICU, Intensive Care Unit.

CI: consistency index, RI: random consistency index that depends on the number of criteria, λ: maximum eigenvector of the matrix, and n: the number of criteria.

Table 2 presents the Analytic Hierarchy Process (AHP) pairwise comparison matrix used to allocate weights for the various criteria related to the COVID-19 Vulnerability Index (CVI). The table showcases the relative importance of each criterion in assessing vulnerability, aiding in the prioritization and decision-making process for strategic interventions and resource allocation.

To ensure the robustness of the statistical analysis, validating the weights assigned using the Analytic Hierarchy Process (AHP) is essential. The Consistency Ratio (CR) plays a crucial role: a CR ≤ 0.1 signifies acceptable consistency, whereas a higher CR suggests the need for review. In this study, a CR value of 0.06 was achieved, indicating that the COVID-19 Vulnerability Index (CVI) criteria matrix is consistent and reliable. ^29^

**Figure 3.**
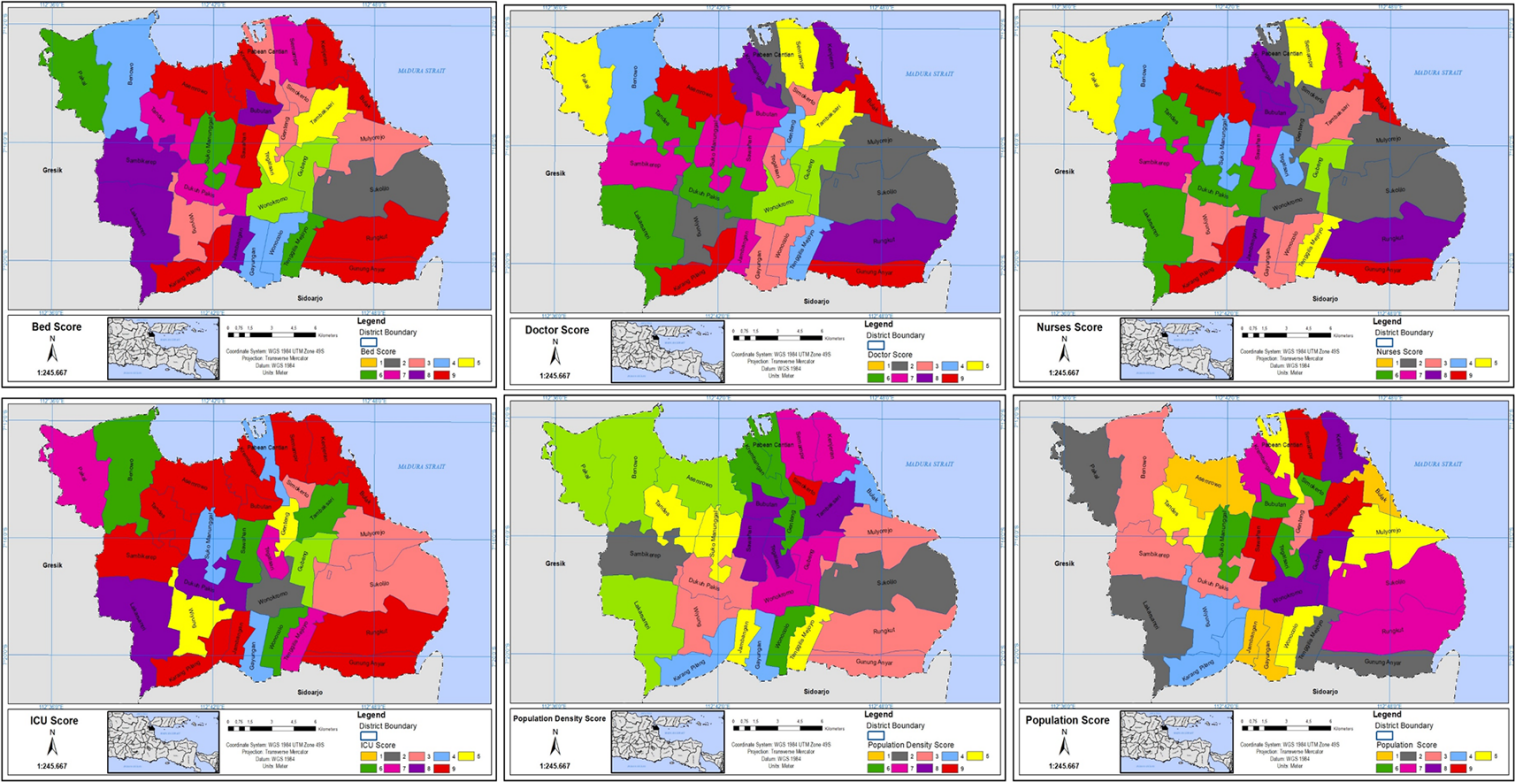
Scored grids of criteria.

In the CVI map, every criterion was categorized into nine value classes, and each class was assigned a score ranging from 1 less important to 9 highly important. ^28^ The chosen criteria were then converted into raster format and reclassified using various GIS tools (see Figure 3).

GIS was utilized to calculate the CVI by employing the weighted overlay summation process. ^29^ This involved aggregating the weighted cell values of various selected criteria. Each criterion’s input layer was multiplied by its respective weight, and the outcomes were combined through summation. In the end, the comprehensive CVI was computed using the natural breaks (Jenks) method in GIS. This CVI value was then employed to create the COVID-19 vulnerability map covering the entirety of the Surabaya city.

$$\mathrm{CVI}=\sum_{i=1}^{n}\mathrm{Wi}\times \mathrm{Sij}$$

**Figure 4.**
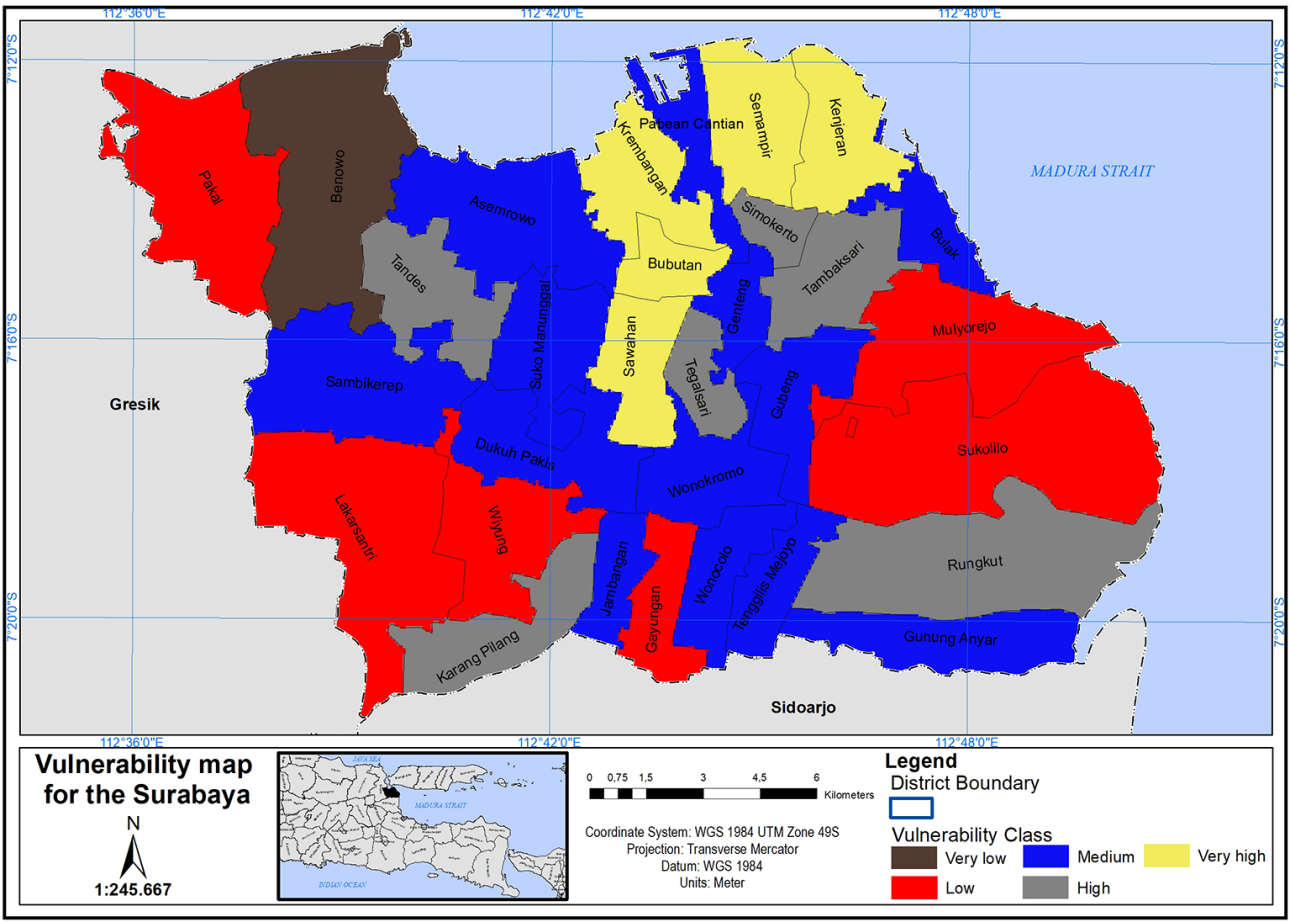
Vulnerability map of Surabaya based on covid-19.

The COVID-19 vulnerability map for the Surabaya was designed (see Figure 4). This map classified the Surabaya districts into five distinct COVID-19 vulnerability categories, ranging from very low to very high. Additionally, Table 3 provides the population counts for each COVID-19 vulnerability class in the Surabaya city.

**Table 3.** Sub-criteria CVI scoring.

No.	Criteria	Sub-criteria	Score
1	Population	42309-49014	1
		49014-56082	2
		56082-61822	3
		61822-70803	4
		70803-88730	5
		88730-99809	6
		99809-116433	7
		116433-161112	8
		161112-219433	9
2	P. Density	2516,19-2890,11	1
		2890,11-3884,37	2
		3884,37-6114,59	3
		6114,59-7656,5	4
		7656,5-11283,61	5
		11283,61-14182,19	6
		14182,19-20154,25	7
		20154,25-27720,78	8
		27720,78-35887,24	9
3	Doctors	3-6	9
		6-16	8
		16-24	7
		24-34	6
		34-59	5
		59-108	4
		108-147	3
		147-210	2
		210-536	1
4	Nurses	5-7	9
		7-22	8
		22-32	7
		32-64	6
		64-137	5
		137-248	4
		248-318	3
		318-1026	2
		1026-1775	1
5	ICU	0	9
		0-2	8
		2-7	7
		7-9	6
		9-11	5
		11-16	4
		16-38	3
		38-69	2
		69-134	1
6	Beds	0	9
		0-40	8
		40-79	7
		79-167	6
		167-236	5
		236-336	4
		336-456	3
		456-659	2
		659-2010	1

## Discussion

Ongoing and resurging diseases that have the potential to become pandemics remain a persistent challenge for nations and healthcare systems, resulting in significant human and economic tolls. This underscores the importance of prioritizing global health readiness in the face of emerging epidemics. Enhancing healthcare infrastructure stands as the most effective safeguard against disease outbreaks and other health-related risks, making it a vital component of health security for all countries. ^30^

In terms of demographic factors, districts like Simokerto, Wonokromo, Gubeng, Sawahan, Tambaksari, Bubutan, Tegalsari, Semampir, and Kenjeran are identified as being in very high to high vulnerability zones. These districts share a high population density. The study specifically selected districts with scores ranging from 7 to 9, which revealed that approximately 47% of Surabaya’s inhabitants are in a high vulnerability zone. On the other hand, related to nurse number to population indicates district of Sambikerep, Sawahan, Kenjeran, Rungkut, Jambangan, Bubutan, Gunung Anyar, Karang Pilang, Asemrowo, Bulak, and Krembangan are under high to very high vulnerable zone. More ever, related to number of doctors to population indicates districts of Jambangan, Sambikerep, Sukomanunggal, Sawahan, Bubutan, Rungkut, Kenjeran, Krembangan, Gunung Anyar, Karang Pilang, Asemrowo, and Bulak are under high to very high vulnerable zone. The criteria of nurses and doctors play a crucial role in responding to an emergency outbreak. However, these factors suffer from a shortage of doctors and nurses to effectively manage any outbreak.

Meanwhile, districts of Dukuh Pakis, Tandes, Semampir, Lakarsantri, Sambikerep, Jambatan Bubutan, Karang Pilang, Gunugng Anyar, Rungkut, Sawahan, Asemrowo, Krembangan, Kenjeraan, and Bulak are under high to very high vulnerable zone because of bed hospital to population in districts. The study selected districts with scores ranging from 7 to 9 only. This revealed that 15 districts, accounting for 47.8% of the total population, have a shortage of hospital beds. This highlights a high vulnerability for the city of Surabaya in the event of a potential emergency outbreak.

However districts of Pakal, Tegalsari, Tenggilis Mejoyo, Lakarsantri, Dukuh Pakis, Karang Pilang, Jambangan, Gunung Anyar, Rungkut, Bulak, Kenjeran, Semampir, Krembangan, Bubutan Asemrowo, Tandes, and Sambilerep are in a highly vulnerable zone due to the ICU capacity in relation to the district’s population. The study selected districts with scores ranging from 7 to 9, revealing that 17 districts have an ICU shortage. This highlights a high vulnerability for the city of Surabaya in the event of a potential emergency outbreak.

The vulnerability map of Surabaya, as depicted in Figure 4 plays a crucial role in assessing the city’s preparedness for a potential emergency outbreak. By identifying districts within Surabaya that exhibit high vulnerability, especially those with high population density and other relevant criteria, this map serves as an essential tool for understanding where vulnerabilities are most pronounced. In the context of a potential emergency outbreak, such as a public health crisis or a natural disaster, areas with high vulnerability, as indicated on the map, may face greater challenges in responding to and managing the crisis effectively. These challenges could include a shortage of healthcare facilities, limited access to medical resources, overcrowding, and socioeconomic factors that hinder residents’ ability to cope with emergencies.

This study proposes a COVID-19 Vulnerability Index (CVI) for Surabaya using GIS-based Multi-Criteria Decision Analysis (MCDA) to integrate demographic, and healthcare factors, identifying high-risk areas to aid resource allocation and targeted interventions. Compared to existing studies that focus on static disaster relief center allocation. Future studies should expand on this research by including dynamic aspects of crisis management and applying GIS and MCDA in various disaster and epidemic contexts, such as evaluating relief centers' efficiency and emergency response performance. ^31^ In addition, this can guide policymakers in resource allocation, enabling strategic planning to target high-risk areas. The findings emphasize the need for improved healthcare infrastructure, better-trained healthcare professionals, and efficient emergency response coordination, ultimately aiding in disaster management and public health preparedness.

## Conclusion

In this research, a vulnerability map was created for the Surabaya using CVI values derived through GIS-based MCDA. Six significant factors were chosen, Weightings for these factors were determined using the AHP pairwise comparison matrix. The GIS was employed to categorize Surabaya’s CVI values into five COVID-19 vulnerability levels: very low, low, medium, high, and very high.

The information provided by this map empowers decision-makers, healthcare professionals, and disaster management teams to allocate resources strategically, implement targeted interventions, and develop comprehensive response strategies tailored to the specific needs of vulnerable districts. By doing so, Surabaya can enhance its resilience and preparedness, ultimately safeguarding the well-being of its residents in the face of potential emergency outbreaks.

## Data Availability

The data for this study owned by the Ministry of Health Republic Indonesia, it can be obtained through the following link; https://sirs.kemkes.go.id/fo/home/profile_rs/1171015. Fighshare: STROBE Checklist for “Mapping vulnerability to potential crisis events in Surabaya city: A GIS-based approach”, https://doi.org/10.6084/m9.figshare.25598073.v1. ^32^ Licence: CC BY 4.0.
