# Supplementary material for: Mapping Vulnerability to Potential Crisis Events in Surabaya City: A GIS-Based Approach
Source: F1000Res. 2024 Jul 5;13:465. Originally published 2024 May 9. [Version 2] doi: 10.12688/f1000research.145182.2 (PMC11333876; doi:10.12688/f1000research.145182.2)
Supplement: Peer review history

# Reviewer report: Reviewer response for version 2

**DOI:** 10.5256/f1000research.168292.r300012

**Published:** 2024-08-19

**Authors:** Subham Roy

**Affiliations:** 1 University of North Bengal, West Bengal, India

ACCEPT

Is the work clearly and accurately presented and does it cite the current literature?

No

If applicable, is the statistical analysis and its interpretation appropriate?

Partly

Are all the source data underlying the results available to ensure full reproducibility?

Yes

Is the study design appropriate and is the work technically sound?

Partly

Are the conclusions drawn adequately supported by the results?

No

Are sufficient details of methods and analysis provided to allow replication by others?

No

Reviewer Expertise:

Spatial analysis, GIS, Remote Sensing

I confirm that I have read this submission and believe that I have an appropriate level of expertise to confirm that it is of an acceptable scientific standard.

# Reviewer report: Reviewer response for version 2

**DOI:** 10.5256/f1000research.168292.r300013

**Published:** 2024-07-22

**Authors:** Peiman Ghasemi

**Affiliations:** 1 University of Vienna, Vienna, Austria

The paper can be indexed in the current form.

Is the work clearly and accurately presented and does it cite the current literature?

No

If applicable, is the statistical analysis and its interpretation appropriate?

No

Are all the source data underlying the results available to ensure full reproducibility?

Yes

Is the study design appropriate and is the work technically sound?

Yes

Are the conclusions drawn adequately supported by the results?

Yes

Are sufficient details of methods and analysis provided to allow replication by others?

Yes

Reviewer Expertise:

Disaster management

I confirm that I have read this submission and believe that I have an appropriate level of expertise to confirm that it is of an acceptable scientific standard.

# Reviewer report: Reviewer response for version 1

**DOI:** 10.5256/f1000research.159093.r276913

**Published:** 2024-06-07

**Authors:** Subham Roy

**Affiliations:** 1 University of North Bengal, West Bengal, India

- Clearly state why Surabaya was chosen for this study. For instance, mention the specific challenges Surabaya faces that make it a critical area for vulnerability mapping. Highlight the expected outcomes of the study, such as identifying high-risk areas for targeted interventions.
- Add overall methodological flowchart for better understanding to the readers.
- Provide specific details on the selection criteria for the six key factors influencing vulnerability. Mention the tools and techniques used in the GIS analysis and how the Analytic Hierarchy Process (AHP) was applied.
- Include specific data points or notable trends found in the results. For example, state the percentage of the city classified as high vulnerability and the key factors contributing to this classification.
- Expand the literature review to include a broader range of studies related to vulnerability mapping and GIS-based MCDA. Discuss different methodologies and their findings, and how they relate to your study.
- Clearly identify the gaps in the existing literature that your study aims to fill. Articulate the novelty and significance of your research objectives. For instance, if previous studies have not focused on the integration of certain criteria or regions, highlight this as a gap your study addresses.
- Include more studies that have used similar methodologies or have focused on similar objectives (Add the literature in table format). Discuss the strengths and limitations of these studies to provide a comprehensive background for your research.
- Critically analyze the existing literature by highlighting the methodologies, findings, and gaps. Explain how your study builds on or diverges from these studies. For example, compare the use of different GIS techniques or criteria in vulnerability mapping.
- Provide a detailed explanation of how the AHP process was applied, including the steps taken to ensure the consistency ratio was ≤0.1. Describe how the pairwise comparison matrix was constructed and used to assign weights.
- Clearly describe the sources of your data, including any databases, surveys, or other sources. Explain how the data was collected and any limitations or biases that may affect the results.
- Elaborate on the GIS techniques used in your study. Explain the weighted overlay summation process and the natural breaks (Jenks) method in more detail. Provide a step-by-step description of how these techniques were applied to create the vulnerability map.
- Present the results with detailed explanations of the tables and figures. For example, explain what Table 2 and Figure 2 show and how they contribute to the overall findings. Provide context for the data presented in these tables and figures.
- Try to validate the results using statistical methods like R2, MAE, RMSE, ROC Curve etc.
- Ensure the statistical analysis is robust and clearly interpreted. Provide more details on the consistency ratio calculation and its significance. Explain how the weights were validated and the implications of the statistical findings.
- Discuss the practical implications of your findings for disaster management and public health preparedness. Provide specific recommendations for policymakers, healthcare professionals, and emergency responders based on your findings.
- Summarize the main findings of the study, highlighting the key contributions to the field. For example, state how the vulnerability map can be used to improve emergency preparedness in Surabaya.
- Emphasize the practical implications of the findings for policymakers and emergency preparedness. For example, discuss how the findings can inform resource allocation and strategic planning

Is the work clearly and accurately presented and does it cite the current literature?

No

If applicable, is the statistical analysis and its interpretation appropriate?

Partly

Are all the source data underlying the results available to ensure full reproducibility?

Yes

Is the study design appropriate and is the work technically sound?

Partly

Are the conclusions drawn adequately supported by the results?

No

Are sufficient details of methods and analysis provided to allow replication by others?

No

Reviewer Expertise:

Spatial analysis, GIS, Remote Sensing

I confirm that I have read this submission and believe that I have an appropriate level of expertise to confirm that it is of an acceptable scientific standard, however I have significant reservations, as outlined above.

## References

[1] : Modeling and mapping geospatial distribution of groundwater potential zones in Darjeeling Himalayan region of India using analytical hierarchy process and GIS technique. *Modeling Earth Systems and Environment* .2022;8(2) : 10.1007/s40808-021-01174-9 1563-1584 10.1007/s40808-021-01174-9

[2] : Urban waterlogging risk as an undervalued environmental challenge: An Integrated MCDA-GIS based modeling approach. *Environmental Challenges* .2021;4: 10.1016/j.envc.2021.100194 10.1016/j.envc.2021.100194

[3] : Flood risk assessment using geospatial data and multi-criteria decision approach: a study from historically active flood-prone region of Himalayan foothill, India. *Arabian Journal of Geosciences* .2021;14(11) : 10.1007/s12517-021-07324-8 10.1007/s12517-021-07324-8

# Response

**Published:** 2024-06-28

**Authors:** Ali Jarghon

**Affiliations:** Public Health Faculty, Airlangga University, surabaya, EastJava, Indonesia

Author response: Thank you so much for your comment, I have corrected/ added upon your request.

 1. Clearly state why Surabaya was chosen for this study. For instance, mention the specific challenges Surabaya faces that make it a critical area for vulnerability mapping. Highlight the expected outcomes of the study, such as identifying high-risk areas for targeted interventions.

**Author response**: Thank you so much for your comment, I have corrected/ added upon your request from **line** 57- 60 in red font

 2. Add overall methodological flowchart for better understanding to the readers.

**Author response**: Thank you so much for your comment, I have corrected/ added upon your request. **Figure 2.**

 3. Provide specific details on the selection criteria for the six key factors influencing vulnerability. Mention the tools and techniques used in the GIS analysis and how the Analytic Hierarchy Process (AHP) was applied.

**Author response**: Thank you so much for your comment, I have corrected/ added upon your request from **line**78-81

 4. Include specific data points or notable trends found in the results. For example, state the percentage of the city classified as high vulnerability and the key factors contributing to this classification.

**Author response**: Thank you so much for your comment, I have corrected/ added upon your request from **line**1(39-142), (153-156)

 5. Expand the literature review to include a broader range of studies related to vulnerability mapping and GIS-based MCDA. Discuss different methodologies and their findings, and how they relate to your study.

**Author response**: Thank you so much for your comment, I have corrected/ added upon your request from **line **36- 49

 6. Clearly identify the gaps in the existing literature that your study aims to fill. Articulate the novelty and significance of your research objectives. For instance, if previous studies have not focused on the integration of certain criteria or regions, highlight this as a gap your study addresses

**Author response**: Thank you so much for your comment, I have corrected/ added upon your request from **line **36-49

 7. Include more studies that have used similar methodologies or have focused on similar objectives (Add the literature in table format). Discuss the strengths and limitations of these studies to provide a comprehensive background for your research.

**Author response**: Thank you so much for your comment, I have corrected/ added upon your request from **line **36-49

 8. Critically analyze the existing literature by highlighting the methodologies, findings, and gaps. Explain how your study builds on or diverges from these studies. For example, compare the use of different GIS techniques or criteria in vulnerability mapping.

**Author response**: Thank you so much for your comment, I have corrected/ added upon your request from **line **36-49

 9. Provide a detailed explanation of how the AHP process was applied, including the steps taken to ensure the consistency ratio was ≤0.1. Describe how the pairwise comparison matrix was constructed and used to assign weights.

**Author response**: Thank you so much for your comment, I have corrected/ added upon your request from **line **93-110

 10. Clearly describe the sources of your data, including any databases, surveys, or other sources. Explain how the data was collected and any limitations or biases that may affect the results.

**Author response**: Thank you so much for your comment, I have corrected/ added upon your request from **line **82

 11. Elaborate on the GIS techniques used in your study. Explain the weighted overlay summation process and the natural breaks (Jenks) method in more detail. Provide a step-by-step description of how these techniques were applied to create the vulnerability map.

**Author response**: Thank you so much for your comment, I have corrected/ added upon your request Summarized in  Figure2

 12. Present the results with detailed explanations of the tables and figures. For example, explain what Table 2 and Figure 2 show and how they contribute to the overall findings. Provide context for the data presented in these tables and figures.

**Author response**: Thank you so much for your comment, I have corrected/ added upon your request from **line **101-110

 13. Try to validate the results using statistical methods like R2, MAE, RMSE, ROC Curve etc.

**Author response**: Thank you so much for your comment, This study utilizes spatial analysis and GIS to predict areas at risk based on collected data. Our approach identifies weaknesses by geographical location, ranking areas from most to least vulnerable according to the weighted results of each parameter. Ultimately, the findings are presented in the form of a map.

 14. Ensure the statistical analysis is robust and clearly interpreted. Provide more details on the consistency ratio calculation and its significance. Explain how the weights were validated and the implications of the statistical findings.

**Author response**: Thank you so much for your comment, I have corrected/ added upon your request from **line **106

 15. Discuss the practical implications of your findings for disaster management and public health preparedness. Provide specific recommendations for policymakers, healthcare professionals, and emergency responders based on your findings.

**Author response**: Thank you so much for your comment, I have corrected/ added upon your request from **line **173- 183

 16. Summarize the main findings of the study, highlighting the key contributions to the field. For example, state how the vulnerability map can be used to improve emergency preparedness in Surabaya.

**Author response**: Thank you so much for your comment, I have corrected/ added upon your request from **line **163- 172

 17. Emphasize the practical implications of the findings for policymakers and emergency preparedness. For example, discuss how the findings can inform resource allocation and strategic planning

**Author response**: Thank you so much for your comment, I have corrected/ added upon your request from **line**173-183

# Reviewer report: Reviewer response for version 1

**DOI:** 10.5256/f1000research.159093.r276920

**Published:** 2024-05-30

**Authors:** Peiman Ghasemi

**Affiliations:** 1 University of Vienna, Vienna, Austria

How did the COVID-19 outbreak evolve into a pandemic, and what were its severe health implications globally?

 Where was the initial COVID-19 case identified, and how quickly did the virus spread to other countries?

 In the introduction, you need to connect the state of the art to your paper goals. Please follow the literature review by a clear and concise state of the art analysis. This should clearly show the knowledge gaps identified and link them to your paper goals. Please reason both the novelty and the relevance of your paper goals. Clearly discuss what the previous studies that you are referring to. What are the Research Gaps/Contributions? Please note that the paper may not be considered further without a clear research gap and novelty of the study.

 Literature Review has the chance to be further improved: it seems that the authors have made the retrospection. However, via the review, what issues should be addressed? What is the current specific knowledge gap? What implication can be referred to? The above questions should be answered. Authors need to propose their study and compare your study with efficient crisis management by selection and analysis of relief centers in disaster integrating GIS and multicriteria decision methods, evaluating the efficiency of relief centers in disaster and epidemic conditions using multi-criteria decision-making methods and GIS: A case study, evaluating the performance of emergency centers during coronavirus epidemic using multi-criteria Decision-making methods, data-driven modeling using system dynamics simulation to provide relief in earthquake based on different scenarios

 What role does population vulnerability play in crisis situations like the COVID-19 outbreak?

 How does social vulnerability contribute to the impact of disasters, and how can government responses exacerbate or alleviate these vulnerabilities?

 What methods are commonly used for vulnerability mapping, especially concerning health-related crises like COVID-19?

 How does Geographic Information Systems (GIS) aid in assessing vulnerability, particularly in the context of epidemic prediction mapping?

 What factors were considered in developing the COVID-19 Vulnerability Index (CVI) for Surabaya, and how were weights assigned to these factors?

 How does the vulnerability map of Surabaya help decision-makers identify and address potential COVID-19 outbreaks, especially in high-risk areas?

Is the work clearly and accurately presented and does it cite the current literature?

No

If applicable, is the statistical analysis and its interpretation appropriate?

No

Are all the source data underlying the results available to ensure full reproducibility?

Yes

Is the study design appropriate and is the work technically sound?

Yes

Are the conclusions drawn adequately supported by the results?

Yes

Are sufficient details of methods and analysis provided to allow replication by others?

Yes

Reviewer Expertise:

Disaster management

I confirm that I have read this submission and believe that I have an appropriate level of expertise to confirm that it is of an acceptable scientific standard, however I have significant reservations, as outlined above.

# Response

**Published:** 2024-06-28

**Authors:** Ali Jarghon

**Affiliations:** Public Health Faculty, Airlangga University, surabaya, EastJava, Indonesia

Author response: Thank you so much for your comments, I have corrected/ added upon your request.

 How did the COVID-19 outbreak evolve into a pandemic, and what were its severe health implications globally?

**Author response:** Thank you so much for your comment, I have corrected/ added upon your request from **line 2-4** in red font

 Where was the initial COVID-19 case identified, and how quickly did the virus spread to other countries?

**Author response:** Thank you so much for your comment, I have corrected/ added upon your request from **line **6-8

 What are the Research Gaps/Contributions?/ Clearly discuss what the previous studies that you are referring to.

**Author response:** Thank you so much for your comment, I have corrected/ added upon your request from **line **43- 49

 What role does population vulnerability play in crisis situations like the COVID-19 outbreak?

**Author response:** Thank you so much for your comment, I have corrected/ added upon your request from **line **21-27

 How does social vulnerability contribute to the impact of disasters, and how can government responses exacerbate or alleviate these vulnerabilities?

**Author response:** Thank you so much for your comment, I have corrected/ added upon your request from **line ** 21-27

 What methods are commonly used for vulnerability mapping, especially concerning health-related crises like COVID-19?

**Author response:** Thank you so much for your comment, I have corrected/ added upon your request from **line **33-37

 How does Geographic Information Systems (GIS) aid in assessing vulnerability, particularly in the context of epidemic prediction mapping        

**Author response:** Thank you so much for your comment, I have corrected/ added upon your request from **line **50- 53

 What factors were considered in developing the COVID-19 Vulnerability Index (CVI) for Surabaya, and how were weights assigned to these factors?

**Author response:** Thank you so much for your comment, I have corrected/ added upon your request from **line **(78-81), (116-121)

 How does the vulnerability map of Surabaya help decision-makers identify and address potential COVID-19 outbreaks, especially in high-risk areas?

**Author response:** Thank you so much for your comment, I have corrected/ added upon your request from **line **163-171

 Authors need to propose their study and compare your study with efficient crisis management by selection and analysis of relief centers in disaster integrating GIS and multicriteria decision methods, evaluating the efficiency of relief centers in disaster and epidemic conditions using multi-criteria decision-making methods and GIS: A case study, evaluating the performance of emergency centers during coronavirus epidemic using multi-criteria Decision-making methods, data-driven modeling using system dynamics simulation to provide relief in earthquake based on different scenarios

**Author response:** Thank you so much for your comment, I have corrected/ added upon your request from **line **173- 183
